# Evaluation of the Adsorption and Desorption Dynamics of Beet Juice Red Dye on Alginate Microbeads

**DOI:** 10.3390/gels8010013

**Published:** 2021-12-24

**Authors:** Anamaria Birkić, Davor Valinger, Ana Jurinjak Tušek, Tamara Jurina, Jasenka Gajdoš Kljusurić, Maja Benković

**Affiliations:** Faculty of Food Technology and Biotechnology, University of Zagreb, 10000 Zagreb, Croatia; anamaria.birkic@gmail.com (A.B.); davor.valinger@pbf.unizg.hr (D.V.); tamara.jurina@pbf.unizg.hr (T.J.); jasenka.gajdos.kljusuric@pbf.unizg.hr (J.G.K.); maja.benkovic@pbf.unizg.hr (M.B.)

**Keywords:** alginate, modeling, adsorption, desorption, beetroot red dye

## Abstract

The use of alginate microcapsules has often been mentioned as one of the ways to remove dyes from waste solvents, water and materials from the food industry. In addition, alginate can be used as a wall material for the microencapsulation of food dyes and their further application in the food industry. The aims of this study were to: (i) determine the effect of the alginate concentration (1, 2, 3 and 4%) on the ability of the adsorption and desorption of natural beetroot red dye and (ii) evaluate the kinetic parameters of the adsorption and desorption process, as well as the factors affecting and limiting those processes. According to the obtained results, the viscosity of alginate solutions increased with an increase in the alginate concentration. Based on *k*_2_ values (the pseudo-second order kinetic rate constant), when a more concentrated solution of alginate was used in the adsorption process, the beads adsorbed a smaller amount of dye. Furthermore, based on the values for *n* derived from the Korsmeyer–Peppas model, the dye release rates (*k*) were higher for beads made with lower alginate concentrations, and this release was governed by a pseudo-Fickian diffusion mechanism (*n* values ranged from 0.2709 to 0.3053).

## 1. Introduction

Encapsulation is defined as a process of confining active compounds within a matrix to achieve one or more desirable effects such as the immobilization, protection, stabilization, controlled release and alteration of a product’s properties [1]. In order to produce microcapsules with desired characteristics, it is necessary to select a suitable microencapsulation method and optimize the process conditions. Nowadays, microcapsules can have a wide variety of functionalities, due to the development of different production technologies and wall materials [2]. The correct selection of wall material is very important, because it affects the efficiency of the microencapsulation process and the stability of the produced microcapsules [3]. Commonly used materials are natural or synthetic polymers such as proteins, carbohydrates and gums. One of the most commonly used polymers is alginate, a natural linear polysaccharide composed of alternating blocks of 1–4 linked-L-guluronic and -D-mannuronic acid residues. Alginates are naturally present in the cell wall of brown algae (Phaeophyceae), and, currently, all commercially available alginate is extracted from algae biomass [4]. It is the most used polymer matrix due to its nontoxicity, biocompatibility and gel formation ability [5]. The microencapsulation of the active ingredient within the protective coating can be performed using different methods. The choice of the most appropriate method depends on the type and properties of the active ingredient, properties of the material used for microencapsulation, particle size required, final use of the microcapsules and cost of the production process [6]. Microencapsulation has found its application in chemical, pharmaceutical, food and textile industries, as well as in the field of environmental protection, where polymer microbeads can be used as adsorbents to remove various impurities from wastewater and waste solvents.

Various natural and synthetic dyes are used in the food industry, with a growing interest in natural dyes. Such a trend is closely related to the perception of synthetic dyes as harmful, while pigments that occur naturally in edible plants are usually considered harmless [7]. Unlike synthetic dyes, natural dye extracts may contain other bioactive ingredients that can improve the functional value of the final product [8]. “Beetroot red” is a natural red food color obtained by extraction from the roots of red beets. It is approved as a food additive in the European Union (EU) and its E number is E162 (betanin). It contains a number of different pigments, all belonging to the group of betalains. Beetroot red is available in liquid or solid form, depending upon the degree of processing, and it is used in a variety of processed foods. There is no indication of intolerance or allergenicity of E162 in the available literature [9]. However, the main disadvantages of natural food dyes are their higher production costs and reduced stability during processing and storage [10]. Since natural dyes show a promising future in food application, but can contain different compounds other than the compounds that are directly responsible for the color formation, their behavior during encapsulation and impact on the adsorption and desorption processes to and from the alginate microbeads have yet to be explored in detail. Furthermore, based on literature data, it is known that different factors such as pH, temperature, initial dye concentration and adsorbent weight can affect the adsorption and desorption of dyes [11,12,13]. In addition, another factor, the influence of which on adsorption and desorption has not been fully defined, is alginate concentration. This factor is of great importance mostly due to the economic feasibility of the process.

Taking into account the abovementioned facts, the aim of this study was to (i) determine the effect of the alginate concentration and adsorbent weight on the ability of the adsorption and desorption of natural beet juice food dye and (ii) evaluate the kinetic parameters of the adsorption and the desorption process, as well as the factors affecting and limiting those processes.

## 2. Results and Discussion

The aim of this research was to determine the influence of the alginate concentration on the adsorption and desorption processes of natural red dye from alginate beads, as well as to determine the kinetic parameters and the limiting factors of the adsorption and desorption processes.

### 2.1. Viscosity of the Alginate Solutions

**Figure 1:**
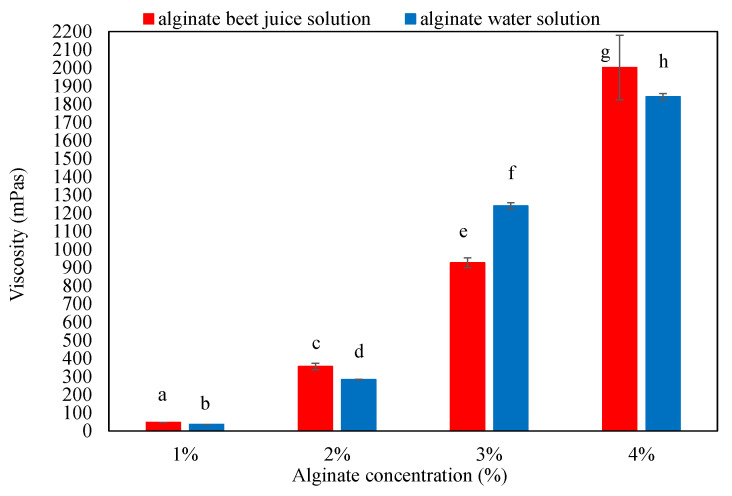
Viscosities of the prepared alginate solutions. Different letters above the bars represent significant differences at *p* < 0.05.

According to literature data, the viscosity of alginate solutions significantly affects beads’ textural properties, which, consequently, also affects their ability to adsorb and release active ingredients [14,15,16]. Viscosities of alginate solutions prepared with distilled water and beetroot juice are shown in Figure 1.

Previous studies have determined that alginate solutions show high viscosity values even at low alginate concentrations (e.g., about 300 mPas for a 2% alginate solution) [17]. The viscosity of the 2% alginate-water solution determined in this study (282.77 ± 1.33 mPas) agrees well with literature data [17] (Figure 1). The results show that, as the concentration of alginate in the solution increased, the viscosity of the solutions increased as well (35.82 ± 0.3 mPas for the 1% solution to 1840.33 ± 17.6 mPas for the 4% alginate solution). The obtained results are consistent with the results of other studies [1,16], in which an exponential increase in viscosity with increasing alginate solution concentration was observed. Previous studies have also determined that the viscosity of the alginate solution must be higher than 60 mPas in order to produce microcapsules with good mechanical properties and the desired spherical shape. In general, alginate solutions with a viscosity above 500 mPas are more difficult to extrude, and the resulting microcapsules often have a deformed shape [16].

The viscosities of alginate–beet juice solutions are also shown in Figure 1. The results show an increase in the measured values of the viscosity with increasing alginate concentrations (from 46.90 ± 0.28 mPas for 1% to 2001.33 ± 177.53 mPas for 4%). It can also be seen that the dye–alginate solutions containing 1%, 2% and 4% alginate were more viscous than alginate solutions of equal concentrations made by dissolving the alginate in distilled water. As a rule, increasing the dry matter content also increased the viscosity [18]. Therefore, alginate solutions prepared by dissolving alginate in beetroot juice generally had higher viscosities than alginate solutions in distilled water due to the higher dry matter content of beetroot juice compared to distilled water.

### 2.2. Diameter, Microstructure and Color of the Microcapsules

**Figure 2:**
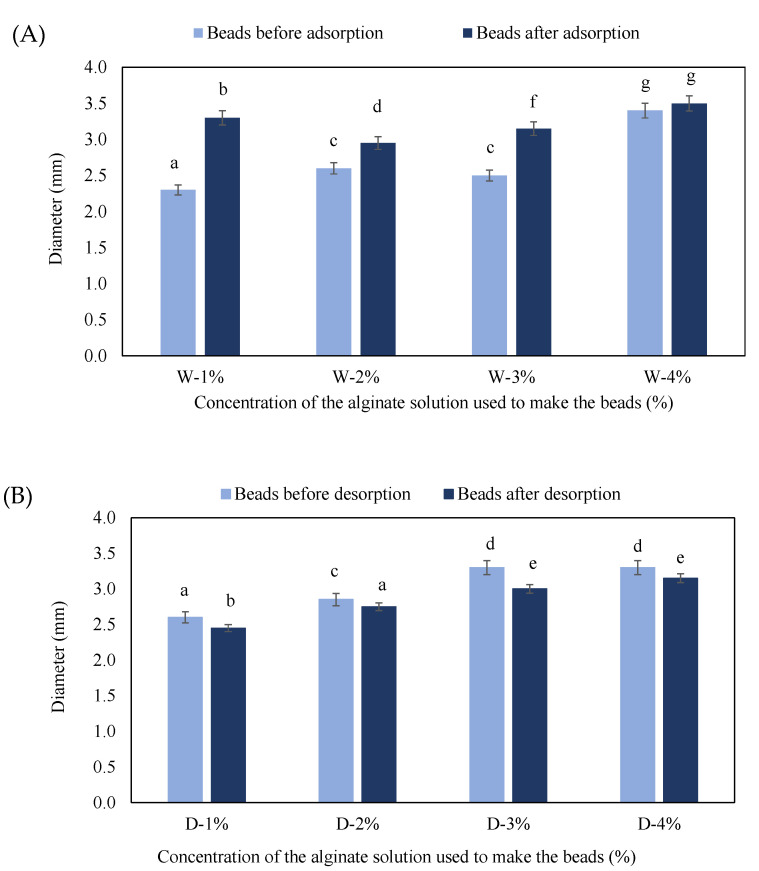
Diameter change of beads before and after the adsorption process (**A**) and before and after the desorption process (**B**). The letter “W” denotes plain beads made with water and subjected to the adsorption process, while the letter “D” denotes beads made with dye and subjected to the desorption process. Different letters (a,b,c,d,e) above the bars represent significant differences at *p* < 0.05.

The diameter change of alginate beads was analyzed before and after adsorption and desorption, and the results are shown in Figure 2.

As seen in Figure 2A, the diameters of prepared microcapsules before adsorption ranged from 2 to 3.5 mm, which agrees well with the results of previous studies [19]. According to some authors, the diameters of the microcapsules is dependent on the speed at which the beads are extruded: the bead diameter decreases with an increasing extrusion rate [5]. It is therefore possible that microcapsules containing lower concentrations of alginate are slightly smaller in diameter compared to those with higher concentrations of alginate due to the higher extrusion rate of the less viscous alginate solution. After the adsorption process, an increase in the diameters of all microcapsules was detected, with the largest change in diameter visible in microcapsules with 1% alginate, while in microcapsules with 4% alginate the change in diameter was the least pronounced. When microcapsules were placed in a dye solution, the concentration of dye molecules outside the microcapsules was significantly higher than the concentration of dye inside them. The concentration gradient caused the diffusion of dye molecules into the beads, resulting in a higher osmotic pressure inside the beads that caused the beads to swell. The reason for the higher swelling of beads with a lower concentration of alginate was the lower degree of crosslinking between the calcium and the alginate, which caused a higher permeability in the surface layer of the bead that made the alginate bead more exposed to environmental influences; thus the diffusion of the surrounding solvent into the bead was easier [20].

**Figure 3:**
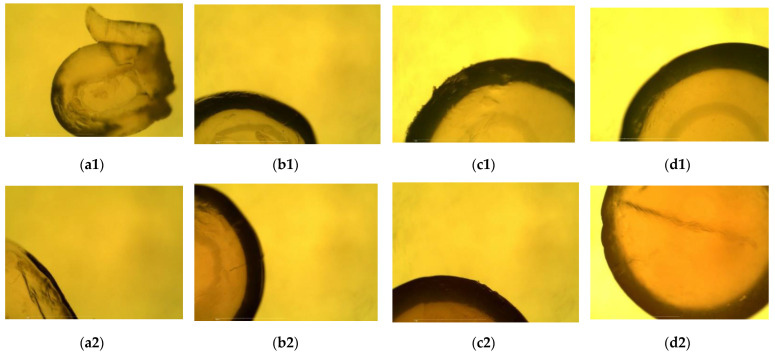
Micrographs of the bead cross-sections before (plain beads made with water) and after the adsorption process (beads with adsorbed dye): 1%, 2%, 3% and 4% plain beads before adsorption (**a1**–**d1**); 1%, 2%, 3% and 4% plain beads after adsorption (**a2**–**d2**).

**Figure 4:**
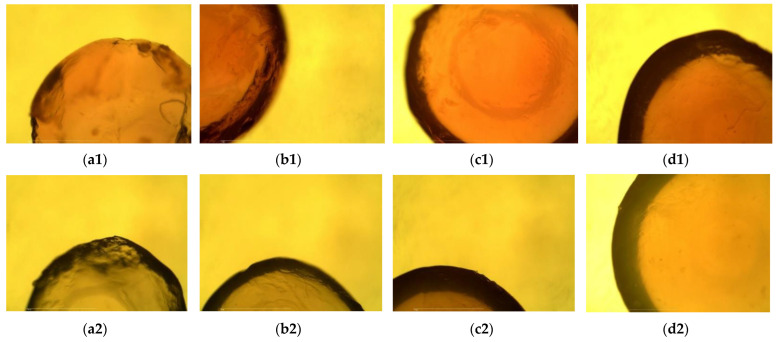
Micrographs of the bead cross-sections before (beads made with dye solution) and after the desorption process (beads after the dye release): 1%, 2%, 3% and 4% dye loaded beads before desorption (**a1**–**d1**); 1%, 2%, 3% and 4% beads after dye desorption (**a2**–**d2**).

Microcapsules prepared with beetroot juice and alginate before the desorption process (Figure 2B) differed in appearance and size. Slightly smaller diameters were measured for microcapsules containing 1% and 2% alginate, compared to microcapsules containing 3% and 4% alginate. Lower-viscosity solutions during the extrusion dripping resulted in microcapsules with smaller diameters in comparison to more viscous solutions, given that the preparation conditions were the same [19]. After desorption, a decrease in the diameter of microcapsules was detected. In this case, no visible trend in the influence of the alginate concentration on the reduction in diameter was noticed. The decrease in diameter was due to the diffusion of color molecules from the inside of the microcapsules into the distilled water due to the high concentration gradient. The appearance of the microcapsules and the changes during the adsorption and desorption processes were also monitored by light microscopy. Micrographs of the beads are shown in Figure 3 and Figure 4.

Before the adsorption process, on the micrographs of the plain beads made with distilled water (Figure 3(a1–d1)), a rougher surface and the irregular shape of the beads made with lower percentages of alginate can be seen. As argued before, solutions containing less alginate were less viscous, and they could be extruded from the syringe at higher rates, which was the cause of these irregular shapes, rougher surfaces and smaller diameters. After the adsorption process, the beads were clearly darker with a slightly reddish tone visible in the micrographs (Figure 3(a2–d2)), which was a result of dye adsorption. Rougher surfaces on the 1% and 2% alginate beads were also seen after the desorption process (Figure 4). The beads made by dissolving the alginate in the dye solution before desorption (Figure 4(a1–d1)) had a more regular shape in comparison to those made only from water, due to the higher viscosity of the dripping solution. Moreover, the lower concentration of alginate in the solution resulted in greater surface roughness. Before the desorption process, the beads were clearly darker compared to the beads after the desorption process (Figure 4 (a2–d2)), which was a confirmation that the dye diffused out of the beads into the surrounding solvent.

Color changes in the beads during adsorption and desorption were monitored using a colorimeter. Color parameters of the beads before and after adsorption are shown in Table 1.

Prior to the adsorption process, the L* value of the plain microcapsules made with lower concentrations of alginate was higher in comparison to those made with higher alginate concentrations. This can be explained by the color of the alginate powder used to make the solutions. Namely, when diluted, the solution exhibited faintly yellow color, which became more intense as the concentration of the solution increased. The color of the microcapsules changed significantly after the adsorption process (Table 1). The L* value of all samples decreased after the adsorption process due to the loss of brightness caused by pigment adsorption from the dye solution. A significant increase in a* values (ranging from green to red) could also be observed, which indicates that the microcapsule had more pronounced red tones after the adsorption process due to betalain adsorption. Namely, the betalains contain two groups of red-purple and yellow pigments, which result in numerous red variations [10]. Values of parameter b* (ranging from blue to yellow), changed from positive to negative in all samples after the adsorption process. Sodium alginate contributed to a higher intensity of yellow in the prepared microcapsules prior to the color adsorption and thus the positive values of parameter b*. A decrease in b* values after adsorption is an indication that red and violet pigments were also adsorbed, causing a shift from positive to negative values. An increase in chroma values was also detected, which indicates a higher degree of color saturation after the adsorption of the color on the alginate microcapsules. In addition, a significant increase in hue was recorded, which indicates more pronounced color tones. The total color change in the microcapsules (Δ*E*), was the largest for microcapsules made of 1% and the smallest for microcapsules made of 4% alginate solution. From the obtained results it can be observed that the total color change of the microcapsules was dependent on the concentration of the alginate used to make the microcapsules: an increasing concentration of the alginate solution also led to a smaller color change.

The color of the microcapsules changed significantly after the desorption process, which is confirmed by the data shown in Table 2. Before desorption, beads with lower alginate concentrations showed lower L* values and higher a*, b*, chroma and hue values. The L* values increased after desorption, meaning that the microcapsules became brighter after the dye was released. The a* values decreased significantly after desorption, indicating a decrease in the intensity of the red color. After desorption, there was an increase in the values of parameter b* in all samples, which indicates less pronounced blue tones in the microcapsules. The value of chroma after color desorption decreased, indicating a lower degree of color saturation and a lower color intensity in the microcapsules. In addition, a slight increase in the value of hue was recorded. The total color change in microcapsules (Δ*E*) was most pronounced in the 1% alginate microcapsules, while the total color change in microcapsules containing higher concentrations of alginate was less pronounced. The total color change in all microcapsule samples after the adsorption and desorption process was greater than 3.0, indicating that the color differences of the microcapsules before and after the processes were visually noticeable to the naked human eye [8].

In the direct comparison of color changes during the adsorption and the desorption process, it can be noticed that the color differences are bigger for the adsorption process. The beads in Table 1 were plain beads, where the color shifted from slightly yellowish to red/purple and therefore showed a bigger change in Δ*E* and hue. On the other hand, during desorption (Table 2), the initial bead color made from beetroot juice was dark red/purple and became lighter red/purple after the desorption process but remained on the same part of the color scale (with no shift from yellow to red) and therefore showed a lower change in Δ*E* and hue. It is important to emphasize that, in this case, this does not mean that the beads made from beetroot juice were faster to absorb and released dye more easily; it only means that they were much darker at the beginning of the desorption process. To confirm the differences in rates of adsorption/desorption, mathematical modeling was required.

### 2.3. Adsorption Dynamics and Modeling

**Figure 5:**
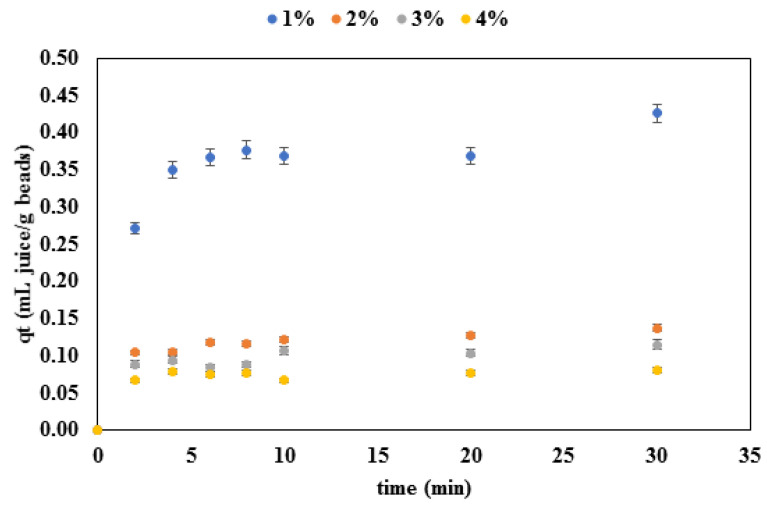
Concentration change dynamics during the adsorption process of the red beetroot dye on alginate microbeads.

The adsorption process of the red beetroot dye on the alginate microcapsules was monitored over 30 min, and the results are shown in Figure 5. The experimental data shown in Figure 5 was then fitted to four different models, and the results are shown in Table 3.

Two different adsorption phases can be distinguished in the adsorption curves shown in Figure 5. The first phase is the phase in which the fast adsorption of the dye from the solution occurred, and it lasted approximately 3 min for beads containing 2%, 3% and 4% alginate and approximately 5 min for the beads with 1% alginate. After the initial fast adsorbing phase, the adsorption slowed down until it reached a steady state at which a balance between the adsorption and release was achieved. Similar results were obtained in other studies that analyzed the adsorption of dyes onto biopolymer and alginate beads: Pradeep Sekhar et al. [21] described the adsorption curve of malachite green as having a smooth initial part and a plateau after the initial rise, while Asadi et al. [22] concluded that the initial adsorption of dye was fast and that the most dye was adsorbed within the first 10 min, after which the curve reached a plateau. This can be explained by the lack of available slots for dye binding as the adsorption progressed. It can also be seen from Figure 5 that the amount of the adsorbed dye was dependent on the alginate concentration of the beads—the amount of adsorbed dye per gram of beads dropped as the alginate concentration increased. In this case, the mass transfer was low due to the high thickness of the boundary layer of microcapsules made with higher alginate contents. Furthermore, previous research also showed that viscosity is one of the critical factors affecting the interphase mass transfer, and that a large solvent viscosity can lead to a significant reduction of the mass transfer coefficient [23]. In this case, a higher viscosity of the alginate solutions affected the texture and diameter of the beads, which, in the end, also affected the amount of the adsorbed dye.

According to the literature data, the adsorption process can be characterized by several models, which include the pseudo-first order, pseudo-second order, Elovich, Avrami and Webber–Morris [24,25] models. The experimental data from this study were fitted to the pseudo-first order, pseudo-second order, Elovich and Webber–Morris models and the results are shown in Table 3. The adequacy of the models was estimated based on the *R*^2^ values and standard errors. For the pseudo first order model, the *R*^2^ values ranged from 0.9604 to 0.9911; for the pseudo-second they ranged from 0.9722 to 0.9928; for the Elovich model they ranged from 0.9821 to 0.9976; and for the Weber-Morris they ranged from 0.6974 to 0.8079. It can be seen that the Elovich and the pseudo-second order model resulted in the best fit with the experimental data; but the pseudo-second order was considered as the more suitable one, since it resulted in much lower standard errors in comparison to the Elovich model. Based on the *k*_2_ values (the pseudo-second order kinetic rate constant), it can be seen that, when a more concentrated solution of alginate was used for the adsorption process, the beads adsorbed a smaller amount of dye, which was a result of a higher mass transfer resistance [23]. In addition, the amount of dye adsorbed at equilibrium (*q_e_*) and the initial adsorption rate (*h*_0_) decreased as the alginate concentration increased. To get a better insight into the diffusion mechanism, the Webber–Morris model was also used. Although the *R*^2^ values for the Webber–Morris model were not as high as for the rest of the models used, it can be seen that the intra-particle diffusion rate (*k_diff_*) decreased for higher concentrations of alginate and that the effect of the boundary layer (*C*) was more pronounced for the lower alginate concentrations. This leads to the conclusion that the adsorption occurred in multiple stages: transfer of the dye from the liquid phase to the bead boundary layer; then diffusion of the liquid through the bead boundary layer; then pore (intra-particle) diffusion of the dye inside the bead. A similar conclusion was reached in previous research dealing with the adsorption of Coomassie brilliant blue R-250 on starch/poly (alginic acid-cl-acrylamide) nanohydrogel [25].

Based on literature data, adsorption is an attractive and favorable technique for dye removal due to its simplicity, low operating cost, high efficiency and low energy consumption [26]. Moreover, the adsorbent can be recovered and reused [22]. However, according to Crini [27], the adsorption process will provide an attractive technology only if the low-cost sorbent is ready for use. In this case, the cost of sodium alginate is rather low (starting from EUR 20 per 1 kg, depending on the purity and intended use), but the adsorbent needs to be prepared prior to the adsorption process. The preparation steps include dissolution of the powder in water, mixing and extrusion through a syringe. All of the preparation steps increase the cost of alginate beads because of the equipment and the manpower required for preparation. As for the potential for the use of alginate in the industrial removal of dyes, studies exist that confirm the feasibility and effectiveness of alginate used in industrial wastewater treatment, but they also emphasize the need for an effective process control, since the adsorption and desorption processes are strongly influenced by temperature, pH, particle size, adsorbent mass and the initial dye concentration [11,12,13]. Alginate is also biodegradable, which can have an adverse effect on long-term applications. These problems can rebut industrial users, as it was the case with chitosan adsorption [28]. Furthermore, Sardar et al. [29] also emphasized the potential of adsorption for dye removal but claim that this process is not transferred fully at pilot as well as industrial scales due to the lack of prediction of the adsorption in some operating conditions, lack of full understanding of the adsorption mechanism and need to develop more low-cost adsorbens.

### 2.4. Desorption Dynamics and Modeling

**Figure 6:**
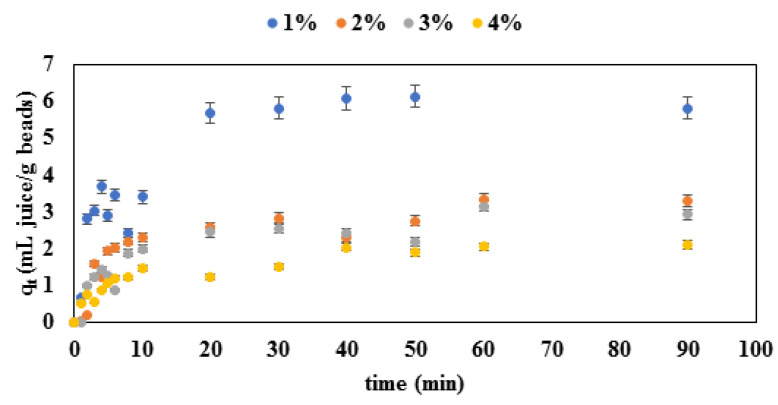
Concentration change dynamics during the desorption process of the red beetroot dye on alginate microbeads.

The concentration change dynamics of the desorption process are shown in Figure 6, and the model parameters obtained from the experimental data are shown in Table 4.

Results shown in Figure 6 describe the dynamics of the release of red beetroot dye from alginate matrix in 2 phases: the first phase, visible during first 10 to 15 min of the process, is a phase where the release from the matrix was fast. After the initial phase, a steady state followed. The steady state phase was reached faster for microcapsules made with lower concentrations of alginate. In addition, the difference in the final amount of the released dye can be seen in the dependence on the alginate concentration of the beads: beads with higher alginate concentrations released a lower amount of dye per gram of beads due to higher mass transfer restrictions. Results shown in Figure 6 are confirmed by the kinetic parameters shown in Table 4. Experimental data were fitted to the first-order, Korsmeyer–Peppas and the Higuchi models. Based on the *R*^2^ values (*R*^2^ > 0.90), it can be concluded that the first-order and the Korsmeyer–Peppas models were both suitable for the description of the release process. It can also be seen that the release rates *(k*) were higher for beads made with lower alginate concentrations. Furthermore, based on the values for *n* derived from the Korsmeyer–Peppas model, it can be concluded that the release is governed by a pseudo-Fickian diffusion mechanism (*n* values ranged from 0.2709 to 0.3053). The Higuchi model was found to be less suitable for the description of the release process due to lower *R*^2^ values (Table 4). A pseudo-Fickian diffusion mechanism of the bioactives from the Lamiaceae plant entrapped in alginate microbeads was also found to describe the release process in a previous research by Benković et al. [30].

## 3. Conclusions

This study deals with the influence of alginate concentration on the adsorption and release profiles of red beetroot dye from alginate microbeads. The findings suggest that alginate beads can be used as an adsorbens to remove dyes from solutions. More importantly, the results suggest that microbeads made with lower concentrations of alginate are more efficient, which is of great importance if the adsorption process is to be implemented for industrial wastewater treatment—lower alginate concentrations also mean lower costs and higher economic feasibility. Furthermore, the importance of the mathematical modeling of the adsorption and desorption processes is clearly indicated—the modeling enables a better insight into the forces that drive the processes and influence the process significantly. It is important to emphasize that, in future research, the effect of the adsorbent mass, pH and initial dye concentration should be explored further, since the knowledge of these influences greatly improves process control, and this is of the utmost importance if adsorption/desorption processes should be implemented in industrial facilities.

## 4. Materials and Methods

### 4.1. Materials

#### 4.1.1. Food Dye

In this work, commercially available beetroot juice produced by dm-drogerie markt GmbH + Co. KG (Karlsruhe, Germany) was used as a natural food dye.

#### 4.1.2. Chemicals

Sodium alginate was purchased from Fisher Scientific (Loughborough, United Kingdom) and anhydrous calcium chloride (CaCl2) was purchased from Gram-Mol d.o.o. (Zagreb, Croatia).

### 4.2. Methods

#### 4.2.1. Preparation of the Dye Solution

Red dye solution was prepared by diluting beetroot juice in distilled water until the absorbance at 482 nm on the spectrophotometer fell below 1.15. The resulting dye solution contained 30% of beetroot juice and 70% distilled water. In all adsorption and desorption experiments beetroot juice solutions of equal concentrations were used.

#### 4.2.2. Preparation of the Alginate Solutions and Microbeads

**Figure 7:**
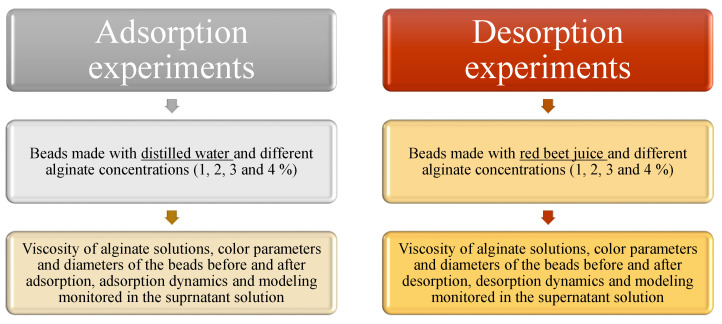
Schematic diagram of the experiment.

Alginate beads were prepared by extrusion dripping technique, according to an experiment scheme shown in Figure 7.

Four different solutions (*w*/*v*) of sodium alginate (1%, 2%, 3% and 4%) were dissolved in 100 mL of distilled water and homogenized for 2 min using a kitchen blender (XB986F Stabmixer, Zentrale Handelsgesellschaft, Offenburg). The solutions were then placed in a refrigerator overnight to remove incorporated air bubbles. A 2% (*w*/*v*) CaCl_2_ receiving solution was also prepared by diluting a known amount of CaCl_2_ in distilled water. Alginate solutions were transferred to a syringe with a medical needle (1.10 × 50 mm) and the alginate solutions were manually squeezed into the CaCl_2_ solution. Produced microcapsules were left in CaCl_2_ solution overnight to stabilize. The microcapsules were then filtered and washed thoroughly with distilled water to remove calcium ion residues from the surface of the beads. Those microcapsules were used for the adsorption experiments.

To ensure that all beads contained the same concentration of dye for the desorption experiments, microcapsules containing red beetroot juice were also prepared. In this case, alginate was dissolved in the previously prepared diluted red beetroot juice instead of distilled water, and the receiving solution was CaCl_2_ (2% *w*/*v*) dissolved in the red beetroot juice instead of water. Those microcapsules were further used for the desorption experiments.

#### 4.2.3. Viscosity of the Alginate Solutions

The viscosity of alginate solutions prior to extrusion dripping was measured using a rotational viscometer Anton Paar QC 300 (Anton Paar, Graz, Austria) at a temperature of 20 ± 2 °C. Viscosity was measured at a rotation speed of 5 rpm using a CC12 sample container. Measurements were performed in triplicate.

#### 4.2.4. Characterization of the Microbeads

##### Diameter

Bead diameters of 10 selected beads prior to and after adsorption were measured using a caliper and the results were presented as mean (*n* = 10) ± standard deviation.

##### Micrographs

Micrographs of the beads were taken using a light microscope (Motic B series, Motic, Barcelona, Spain) at 4× magnification, coupled with a Moticam 3 series camera with a CMOS sensor, a 16 mm focusable lens and a 3 MB capture resolution. The microscales were added to the micrographs using the built-in Motic Images Plus v.2.0. software (Moticam, Barcelona, Spain).

##### Color Measurement

The color of the beads was measured using the PCE-CSM3 colorimeter (PCE Instruments, Germany) with prior white plate calibration. Five color parameters were determined (Hunter’s color coordinates): L* (lightness), a* (represents the range from green to red), and b* (represents the range from blue to yellow), chroma (represents relative saturation) and hue (represents angle of the hue). To describe the color changes during the adsorption and desorption process of the beads, the total color change (Δ*E*) was determined, according to Equation (1):

(1) $$\Delta E=\sqrt{{\left(L^{*}-L_{0}\right)}^{2}+{\left(a^{*}-a_{0}\right)}^{2}+{\left(b^{*}-b_{0}\right)}^{2}}$$

where L*, a* and b* were determined after adsorption/desorption, while *L*_0_, *a*_0_ and *b*_0_ were determined before the adsorption/desorption. Three parallel measurements were performed for each sample, and the results are presented as the mean value ± standard deviation.

#### 4.2.5. Spectrophotometric Determination of Dye Concentration in the Supernatant

The concentration of dye in the supernatant was determined spectrophotometrically using UV–vis spectrophotometer Biochrom Libra S12 (Biochrom, UK) at the wavelength of 482 nm. For the calibration curve, samples of beetroot juice of different dilutions (in the range of 60 to 400) were prepared by pipetting certain volumes of beetroot juice and distilled water into 2 mL plastic tubes. The concentration of dye in samples was determined based on the obtained calibration curve. Measurements were performed in triplicate.

#### 4.2.6. Adsorption Experiments

Adsorption experiments were performed by placing 1000 plain microbeads into a tempered (*T* = 30 °C) red beetroot juice solution (*V* = 120 mL). When the microbeads were placed into the dye solution, the stopwatch was started. Supernatant samples (*V* = 800 µL) were taken from the reaction mixture at regular time intervals of 0, 2, 4, 6, 8, 10, 15, 20 and 30 min. Adsorption experiments were performed separately on microcapsules produced from different concentrations of alginate solution (a total of four adsorption experiments, one for each initial alginate concentration).

#### 4.2.7. Desorption Experiments

A total of 1000 alginate beads made with red beetroot juice were added to 120 mL of distilled water, previously tempered at 30 °C. At the moment when the beads were added to the distilled water, the stopwatch was started. Supernatant samples (*V* = 800 µL) were taken from the reaction mixture at regular time intervals of 0, 1, 2, 3, 4, 5, 6, 8, 10, 20, 30, 40, 50, 60 and 90 min. Desorption experiments were performed separately on microcapsules produced from different concentrations of alginate solution (a total of four desorption experiments, one for each initial alginate concentration).

#### 4.2.8. Statistical Analysis and Modelling

Basic statistical analysis including average values and standard deviations of parallel measurements was performed using Microsoft Excel software system v.2105 (Microsoft Corporation, Redmond, DC, USA), while significant differences between samples were assessed using *t*-test for independent samples in Statistica v. 14 (Tibco Software Inc., Palo Alto, CA, USA).

For estimation of kinetic parameters for adsorption, the amount of adsorbate adsorbed on the beads was calculated according to Equation (2):

(2) $$q_{t}=\frac{\left(c_{0}-c_{t}\right)\cdot V}{m}$$

where *q_t_* is the amount of adsorbed solute (adsorbate) at time *t* (mL juice/g beads), *c*_0_ is the concentration of the solution at time *t* = 0 (mL/mL), *c_t_* is the concentration of the solution at time *t* (mL/mL), *V* is the volume of the adsorbate solution (mL) and *m* is the mass of the adsorbents (beads; g).

Three models were used to fit the experimental data (pseudo first-order, pseudo second-order and Elovich (Equations (3)–(5)). Furthermore, the effect of intraparticle diffusion and boundary layer on the adsorption process was estimated using the Webber–Morris model (Equation (6)):

(3) $$q_{t}=q_{e}\left(1-\exp\left(-k_{1}t\right)\right)$$

(4) $$q_{t}=\frac{k_{2}\cdot q_{e}^{2}\cdot t}{1+k_{2}\cdot q_{e}\cdot t}$$

(5) $$q_{t}=\frac{1}{\beta }\ln\left(1+\alpha \beta t\right)$$

(6) $$q_{t}=K_{diff}\cdot t^{0.5}+C$$

where *q_e_* represents the amount of adsorbed solute at equilibrium (mL juice/g beads); *q_t_* the amount of adsorbed solute at time *t* (mL juice/g beads); *t* represents time (min); *k*_1_ is the pseudo first-order rate constant (min^−1^); *k*_2_ is the pseudo-second order kinetic rate constant (g beads/mL juice min); α (mL juice/g min) and *β* (g beads/mL juice) are the Elovich constants; *K_diff_* is the intraparticle diffusion rate constant (mL juice/g beads min^0.5^); and *C* is the intercept, which indicates the thickness of the boundary layer (mL juice/g beads). From the data obtained from Equations (2) and (3), the initial rate of adsorption (mL juice/g min) *h*_0_ could be calculated from Equations (7) and (8):

(7) $$h_{0}=k_{1}\cdot q_{e}$$

(8) $$h_{0}=k_{2}\cdot q_{e}^{2}$$

where *k*_1_ and *k*_2_ are the pseudo first-order and pseudo second-order kinetic rate constants, while *q_e_* is the amount of adsorbed solute calculated from the pseudo first-order or pseudo second-order kinetic model, respectively.

For estimation of kinetic parameters for desorption, three models were used: first-order kinetic model, Korsmeyer–Peppas model and the Higuchi model (Equations (9)–(11)):

(9) $$q_{t}=q_{0}\cdot e^{-kt}$$

(10) $$q_{t}=k\cdot t^{n}$$

(11) $$q_{t}=k\cdot t^{0.5}$$

where *q_t_* represents the amount of released dye at time *t* (mL juice/g beads), *t* represents time (min), *q*_0_ represents the amount of released dye at time *t* = 0 (mL juice/g beads), *k* represents the release rate (min^−1^) and *n* is the release exponent that describes the release mechanism: *n* < 0.5 indicates a pseudo-Fickian diffusion mechanism; *n* = 0.5 indicates a Fickian mechanism; 0.5 < *n* < 1 indicates an anomalous diffusion mechanism; and *n* = 1 indicates a non-Fickian diffusion mechanism [14]. The Statistica v.14 software package (Tibco Software Inc., Palo Alto, CA, USA) was used for model parameter estimation, with a non-linear estimation using the Levenberg–Marquardt algorithm with maximum of 500 iterations and convergence criterion of 10^−6^.

## Figures and Tables

**Table 1:** Color parameters of the beads before and after adsorption. Different letters (a,b,c,A,B,C) in the superscript in the same column represent significant differences at *p* < 0.05. (*—all samples before and after adsorption were statistically significantly different at *p* < 0.05).

Alginate Concentration [%]	L*		a*		b*		Chroma		Hue		Δ*E*
	Before *	After *	Before *	After *	Before *	After *	Before *	After *	Before *	After *	
1	57.26 ± 0.78 ^a^	32.95 ± 0.87 ^A^	3.07 ± 0.11 ^a^	38.65 ± 1.29 ^A^	4.70 ± 0.16 ^a^	−2.53 ± 0.07 ^A^	5.61 ± 0.19 ^a^	38.73 ± 1.29 ^A^	57.08 ± 0.54 ^a^	356.29 ± 0.05 ^A^	43.69 ± 1.19 ^a^
2	57.62 ± 1.34 ^a^	30.07 ± 0.16 ^B^	2.80 ± 0.07 ^b^	26.55 ± 0.06 ^B^	4.61 ± 0.06 ^a^	−7.02 ± 0.24 ^B^	5.40 ± 0.09 ^a^	27.47 ± 0.11 ^B^	58.72 ± 0.26 ^b^	345.19 ± 0.46 ^B^	38.19 ± 1.20 ^b^
3	55.83 ± 0.87 ^b^	38.11 ± 1.82 ^C^	3.17 ± 0.11 ^c^	27.27 ± 1.20 ^B^	4.36 ± 0.11 ^b^	−11.16 ± 0.36 ^C^	5.39 ± 0.09 ^a^	29.47 ± 1.25 ^C^	53.99 ± 1.35 ^c^	337.73 ± 0.24 ^C^	33.71 ± 1.47 ^c^
4	54.61 ± 1.75 ^b^	36.63 ± 0.23 ^C^	3.02 ± 0.41 ^a^	25.44 ± 0.14 ^C^	3.79 ± 0.24 ^c^	−10.28 ± 0.03 ^D^	4.85 ± 0.43 ^b^	27.44 ± 0.13 ^B^	51.58 ± 2.25 ^d^	338.00 ± 0.15 ^C^	32.00 ± 1.56 ^c^

**Table 2:** Color parameters of the beads before and after desorption. Different letters (a,b,c,A,B,C) in the superscript in the same column represent significant differences at *p* < 0.05. (*—all samples before and after desorption were statistically significantly different at *p* < 0.05).

Alginate Concentration [%]	L*		a*		b*		Chroma		Hue		Δ*E*
	Before *	After *	Before *	After *	Before *	After *	Before *	After *	Before *	After *	
1	30.75 ± 0.59 ^a^	49.10 ± 1.48 ^A^	31.52 ± 1.53 ^a^	11.61 ± 0.38 ^A^	−9.41 ± 2.09 ^a^	−0.14 ± 0.12 ^a^	32.88 ± 0.82 ^a^	11.61 ± 0.37 ^A^	343.85 ± 5.00 ^a^	359.30 ± 0.60 ^A^	28.62 ± 2.46 ^a^
2	31.23 ± 0.41 ^a^	40.43 ± 1.18 ^B^	29.63 ± 0.56 ^b^	13.27 ± 0.09 ^B^	−9.69 ± 0.23 ^a^	−4.17 ± 0.29 ^b^	31.16 ± 0.53 ^b^	13.90 ± 0.06 ^B^	341.88 ± 0.59 ^a^	342.57 ± 1.24 ^B^	19.56 ± 0.91 ^b^
3	32.02 ± 1.64 ^a^	44.16 ± 0.39 ^C^	25.32 ± 0.72 ^c^	12.46 ± 0.16 ^C^	−11.57 ± 0.37 ^b^	−3.83 ± 0.15 ^c^	27.72 ± 0.52 ^c^	13.04 ± 0.11 ^C^	335.32 ± 0.88 ^b^	342.90 ± 0.85 ^B^	19.30 ± 1.39 ^b^
4	34.36 ± 0.10 ^b^	48.55 ± 0.44 ^A^	26.14 ± 0.06 ^d^	10.59 ± 0.23 ^D^	−11.79 ± 0.14 ^b^	−3.73 ± 0.22 ^c^	28.68 ± 0.10 ^d^	11.23 ± 0.17 ^A^	335.74 ± 0.22 ^b^	340.59 ± 1.34 ^C^	22.54 ± 0.39 ^c^

**Table 3:** Parameters of the adsorption process. Values in brackets represent standard errors of parameters estimates. Values with an asterisk (*) represent significant values at *p* < 0.05.

Model/Parameter	1%	2%	3%	4%
**Pseudo first-order**				
*q_e_* (mL juice/g beads)	0.3847 * (0.0093)	0.1229 * (0.0040)	0.0991 * (0.0046)	0.0759 * (0.0019)
*k*_1_ (min^−1^)	0.5962 * (0.0825)	0.8056 * (0.1930)	1.0702 (0.5008)	1.1264 * (0.2973)
*h*_0_ (mL juice/g min)	0.2293 *	0.0990 *	0.1060	0.0855 *
*R* ^2^	0.9911	0.9814	0.9604	0.9885
**Pseudo second-order**				
*q_e_* (mL juice/g beads)	0.4164 * (0.4164)	0.1321 * (0.0040)	0.1064 * (0.0062)	0.0777 * (0.0029)
*k*_2_ (g beads/mL juice min)	2.4856 * (0.6008)	10.7692 * (3.1204)	16.2162 (10.3003)	53.0777 (42.5794)
*h*_0_ (mL juice/g min)	0.4310 *	0.1879 *	0.1836	0.3204
*R* ^2^	0.9928	0.9926	0.9722	0.9869
**Elovich**				
*β* (g beads/mL juice)	22.7399 * (5.0187)	81.3751 * (9.4447)	103.9401 * (34.9979)	115.5526 * (39.8251)
*α* (mL juice/g min)	19.8904 (31.8755)	13.6063 (23.6063)	28.9798 (28.9798)	4.6740 (11.6083)
*R* ^2^	0.9886	0.9976	0.9821	0.9869
**Webber–Morris**				
*K_diff_* (mL juice/g beads min^0.5^)	0.0634 * (0.0189)	0.0204 * (0.0061)	0.0168 * (0.0051)	0.0109 (0.0046)
*C* (mL juice/ g beads)	0.1430 (0.0597)	0.0481 * (0.0192)	0.0392 (0.0162)	0.0355 * (0.0145)
*R* ^2^	0.8079	0.8077	0.8006	0.6974

**Table 4:** Parameter estimates for the desorption process. Values in brackets represent standard errors of model estimates. Values with an asterisk (*) represent significant values at *p* < 0.05.

Model/Parameter	1%	2%	3%	4%
**First-order**				
*q*_0_ (mL juice/g beads)	5.0240 * (0.5113)	3.4217 * (0.3173)	2.7526 * (0.2396)	1.6915 * (0.1335)
*k* (min^−1^)	0.1145 * (0.0276)	0.1399 * (0.0283)	0.0997 * (0.0217)	0.0633 * (0.0145)
*R* ^2^	0.9319	0.9239	0.9328	0.9317
**Korsmeyer–Peppas**				
*k* (min^−1^)	2.0420 * (0.2944)	0.9548 * (0.1786)	0.8130 * (0.1346)	0.6286 * (0.0636)
*n*	0.2709 * (0.0431)	0.2915 * (0.0534)	0.3053 * (0.0469)	0.2834 * (0.0290)
*R* ^2^	0.9256	0.9022	0.9261	0.9643
**Higuchi**				
*k* (min^−1^)	0.9063 * (0.0792)	0.4458 * (0.0372)	0.3990 * (0.0296)	0.2858 * (0.0192)
*R* ^2^	0.7523	0.7900	0.8337	0.8328

## Data Availability

Not applicable.
